# IFNγ synergies with cold atmospheric plasma in triggering colorectal cancer cell ferroptosis via the IFNγ/IFNR2/APC/TCF4/GPX4 axis

**DOI:** 10.18632/aging.204985

**Published:** 2023-09-04

**Authors:** Xinyu Lv, Fu-le He, Yilin Dai, Xiaofeng Dai

**Affiliations:** 1 Wuxi School of Medicine, Jiangnan University, Wuxi 214122, Jiangsu, China; 2 Zhejiang Chinese Medicine Museum, Zhejiang Chinese Medical University, Hangzhou 310053, Zhejiang, China; 3 National Local Joint Engineering Research Center for Precision Surgery and Regenerative Medicine, Shaanxi Provincial Center for Regenerative Medicine and Surgical Engineering, First Affiliated Hospital of Xi’an Jiaotong University, Xi’an 710061, Shaanxi, China

**Keywords:** IFN-γ, colorectal cancer, stem cell, ferroptosis

## Abstract

Colorectal cancer accounts for the second most common cancer-related lethality. Intestinal stem cells are responsible for enteric homeostasis maintenance that, once being transformed, become colorectal cancer stem cells. Arresting cancer stemness represents an innovative strategy for colorectal cancer management. Using intestinal stem cell organoids as the primary model, we screened common inflammatory cytokines to identify key players targeting cancer stemness. We also explored the downstream signaling that drives the functionalities of the identified cytokine through both experimental investigations and computational predictions. As the results, we identified IFNγ as the key cytokine capable of arresting intestinal stem cells via the IFNγ/IFNGR2/APC/TCF4/GPX4 axis, proposed its role in killing colorectal cancer stem cells via triggering GPX4-dependent ferroptosis, and demonstrated its synergistic anti-cancer effect with cold atmospheric plasma in killing colorectal cancer cells that is worthy to be experimentally validated.

## INTRODUCTION

Colorectal cancer (used to describe cecum, colon, rectal cancers, and sometimes abbreviated as colon cancer) is the second most common cause of cancer death in the United States following lung cancer [1], with the estimated deaths being 52550 cases in the USA in 2023 [2]. Accumulating evidence has supported the concept that cancer stem cells (CSCs) play central roles in the initiation of malignant tumors including colorectal cancers. Colorectal CSCs are transformed crypt base columnar intestinal stem cells (ISCs), with Lgr5^+^ (leucine-rich repeat-containing G protein-coupled receptor 5) being the characterization marker of both colorectal CSCs and ISCs [3, 4]. ISCs are capable of regenerating all epithelial cells in the small intestine (SI) and large intestine (LI) [5], and considered the origin of colorectal cancers driving cell to self-renew under both pathological and physiological conditions [5, 6]. Paneth cells are progeny of ISCs that provide an epithelial niche for Lgr5^+^ ISCs in SI [7, 8], and specific activation of β-catenin signaling in Lgr5^+^ ISCs is a known cause of adenoma [6].

Colorectal CSCs have been shown to be associated with enhanced Wnt activation [9] or cytokine regulation [10]. The inflammatory cytokine interferon-γ (IFNγ) is a known cause of Paneth cell niche damage [11–13]. Enhanced IFNγ gene signature (*IFNγ, CD274, LAG3, CXCL9*) is associated with higher overall response rates and longer median progression-free survival among metastasized non-small cell lung carcinoma, urothelial cancer or melanoma patients receiving PD-1 inhibitors or anti-PD-L1 antibodies [14, 15]. These evidences collectively suggest the possible clinical benefits of IFNγ in cancer treatment that have been increasingly acknowledged.

Despite intensive efforts having been devoted to uncover the impacts of IFNγ signaling and cytotoxicity on ISC compartment and colorectal CSC damage, the spectrum of IFNγ targets is far from complete. Through *in vitro* and *ex vivo* organoid modeling, we identified, in this study, APC as a novel target of IFNγ that mediated its suppressive role on the proliferation of ISCs and its transformed peers.

We, in addition, demonstrated the efficacy of cold atmospheric plasma (CAP), a redox regulatory tool and an emerging onco-therapeutic with selectivity against cancer cells, in synergizing with IFNγ towards the trigger of colorectal cancer cell ferroptosis via the IFNγ/IFNR2/APC/TCF4/GPX4 axis that may represent an innovative remedy for colorectal cancer treatment.

## MATERIALS AND METHODS

### Plasma source

A home-made CAP source (Supplementary Figure 1) was used in this study, which is composed of a controlled power supply, helium (He) gas cylinder, rotameter and plasma jet. The peak-to-peak voltage applied to the electrode was set to the range of 0.96–1.24 KV, the sine wave frequency was set at 10 kHz, the flow rate of He was set at 1 L/min, and the distance between the CAP source and the dielectric surface was fixed to 13 mm. Plasma activated medium (PAM) was prepared by setting the distance between the CAP nozzle and the media surface to 13 mm, the peak-to-peak electrode voltage to 1.1 KV, the sine wave frequency to 8.8 KHz, the He gas flow rate to 1 L/min. In each assay, 2 mL of the cell culture medium in a 12-well plate was exposed to CAP for 3 min, and CAP-treated group was obtained by replacing cell culturing medium with PAM.

### Cell culture

Colon carcinoma cells HCT8, purchased from Zhejiang Meisen Technology, were cultured using RPMI1640 (#SH30809.01, Hyclone) supplemented with 10% FBS (#12B196, ExCell Bio), and incubated under 37°C, 5% CO_2_.

### Organoid culture

Organoids were cultivated following the protocol provided in [16]. Cecum, colon, stomach, liver and pancreas tissues from C57 mouse model aged 6~8 weeks were dissociated and cultivated using the corresponding organoid cultures for organoid isolation. Tissues were dissociated using 10 ml organoid medium (cultivating media and supplemented cytokines are listed in Supplementary Table 1). The tissues were cut with scissors and incubated using 1:10 dilution of collagenase/hyaluronidase (#07919, StemCell™ Technologies) at 37°C for 15 min. The dissociated tissue was rotated at 350 g for 5 min, re-suspended using 5 ml PBS (#BL302A, Biosharp) and rotated again. The tissue was trypsinized using 10 ml TrypLE™ Express (#12604013, ThermoFisher) and incubated at the room temperature for 5 min. Trypsinization was terminated by adding 10 ml modified Hank’s balanced salt solution (HBSS; #H1025, Solarbio) supplemented with 5% CS-FBS, 10 μM Y-27632 and 100 μg/ml Primocin, followed by centrifugation at 400 g for 5 min. The dissociated cell clusters (about 2–10 cells per cluster, and 1 × 10^6^ cells in total) were filtered using 100 μM cell filter (#352350, Corning), rotated and re-suspended using 60% Matrigel (#3432-005-01, Biotechne). 25 μl aforementioned mixture of cell clusters and Matrigel were added to each hole of a 6-well plate (#3736, Corning), and the plate was incubated at 37°C and 5% CO_2_ for 30 min for solidification. After forming solid droplets, 1.5 ml organoid medium was added to each hole, and the medium was refreshed every 3–4 days.

For organoid passage, 1 mg/ml disperse enzyme (#07913, StemCell Technologies) was added to the culture medium followed by incubation at 60°C for 37 min. After digesting Matrigel, the organoids were subsequently centrifuged at 350 g for 5 min, washed in PBS, and centrifuged again under the same parameter setting. The organoids were supplemented with 5 ml TrypLE Express, incubated at the room temperature for 3 min, and then mechanically dissociated into small cell clusters through liquid transfer. Organ-like organs were sub-cultured at 1:2 or 1:3 dilution rates every 1–2 weeks.

### Plasmids and transfection

Plasmids for generating p53 knockout (p53-KO), APC knockout (APC-KO), and Kras over-expression (Kras-OE) mutants were purchased from YunzhouBio (Guangzhou, China) and transfected into organoids by electroporation using nuclear transfection instrument (#4D-Nucleofector, Lonza). The organoids were subcultured using the organoid culture medium without antibiotics 2 days prior to electroporation. The organoids were dissociated into clusters of 10–15 cells, resuspended using electroporation buffer containing electroporation enhancer, and then mixed with 10 μl of 50 mM plasmids. The mixture was transferred to a pre-cooled 2 mm electroporation cuvette. The mixture was transferred to a pre-cooled 2 mm electroporation cuvette. Electroporation was performed according to the configured program (voltage: 1.5 kV; time: 8.0 msec). After electroporation, pre-heated culture medium was immediately added to the electroporation colorimetric dish, and the mixture was incubated at 37°C for 40 min. RNP complex electroporated Organoid was used as the negative control. The knockout and over-expression efficiencies were measured using protein western blotting.

### Quantitative real-time PCR (qRT-PCR)

RNA was extracted according to instructions of the VAZYME Cell RNA Extraction Kit (#RC112, Vazyme). The extracted RNA and the system required for qRT-PCR assays were reversely transcribed following instructions of the Takara reverse transcription kit (#RR037, Takara). Information on primers used in this study was summarized in Supplementary Table 2, and qRT-PCR assays were performed using the Roche LightCycler 480. The relative mRNA expression levels were calculated by the 2^−ΔΔCt^ method. The reagents used to process cells are listed in Supplementary Table 3 and inhibitors used are listed in Supplementary Table 4.

### Western blot

The total proteins were extracted from tissues and cells using RIPA (#P0013B, Beyotime) supplemented with protease and phosphatase inhibitors (#P1005, Beyotime). The concentration of extracted proteins was determined using the BCA method (#P0010, Beyotime). Extracted proteins were separated by 10% SDS-PAGE and transferred to poly (vinylidene fluoride) (PVDF) membranes (#IPVH00010, Millipore). The membranes were blocked using the blocking buffer (5% evaporated milk) and incubated using the indicated primary antibody overnight at 4°C. The membranes were incubated using horseradish peroxidase (HRP) conjugated secondary antibodies for 1 h at the room temperature and washed using tris-buffered saline and Tween 20 (TBST). The western blots were developed using enhanced chemiluminescence (#E412-02, Vazyme). The antibodies used are listed in Supplementary Table 5.

### Glutathione (GSH) assay

Appropriate amount of cells were inoculated in 6-well plates followed by incubation at 37°C overnight. Cells were treated with IFNγ or IFNα, and cultured for 24 h. Cells were collected and supplemented with the freshly prepared protein remover M solution. Samples were rapidly frozen and thawed twice using liquid nitrogen and 37°C water bath. Samples were put in a 4°C refrigerator standstill for 5 min, followed by centrifugation at 10000 g for 10 min. The supernatant was collected and the total glutathione was determined. The total glutathione detection working solution was prepared following the manufacture’s protocol, and the standard curve was drawn by diluting the standard sequentially according to a pre-designed concentration gradient. The sample and total glutathione detection working solution were added into a 96-well plate in order, mixed well, and incubated at the room temperature for 5 min. 50 μL of 0.5 mg/mL reduced nicotinamide adenine dinucleotide phosphate (NADPH) solution was added to each well, and the total amount of glutathione in each well was determined using the microplateReader (#Epoch, BioTek) according to the absorbance and the standard curve.

### Lipid peroxidation level determination assay

Appropriate amount of HCT8 cells were inoculated into a 12-well plate and incubated overnight at 37°C and 5% CO_2_. Cells were treated with IFNγ, CAP, and IFNγ + CAP followed by cultivation at 37°C and 5% CO_2_ for 24 hours. Culture medium was refreshed and MBODIOY-C11 was added to make its working concentration at 5 uM. Cells were incubated in the darkness at 37°C and 5% CO_2_ for 30 min before being photographed using a fully automatic upright fluorescence microscope (# Axio Imager Z2, ZEISS).

### Cytokine screening assay

5 ng/ml IL-1α, IL-1β, IL-2, IL-3, IL-4, IL-5, IL-6, IL-7, IL-9, IL-10, IL-11, IL-12, IL-13, IL-15, IL17A, IL17F, IL21, IL22, IL25, IL31, IL33, IFNα, IFNβ, IFNλ2, Amphiregulin, GM-CSF, 10 ng/ml TSLP, and 1 ng/ml and 5 ng/ml IFNγ were used for screening the key cytokine inhibiting the growth of organoids under study. Cytokines were co-incubated with organoids for 24 hours. RNA was extracted for qRT-PCR analysis.

### Signaling inhibition assay

20 μM Z-VAD-FMK, 10 μM Z-DEVD-FMK, 10 μM GSK872, 5 μM Y27632, and 10 μM YVDG were co-cultivated with organoids for 24 hours before performing downstream assays.

## RESULTS

### IFNγ is the key cytokine inhibiting SI spheroid growth

**Figure 1:**
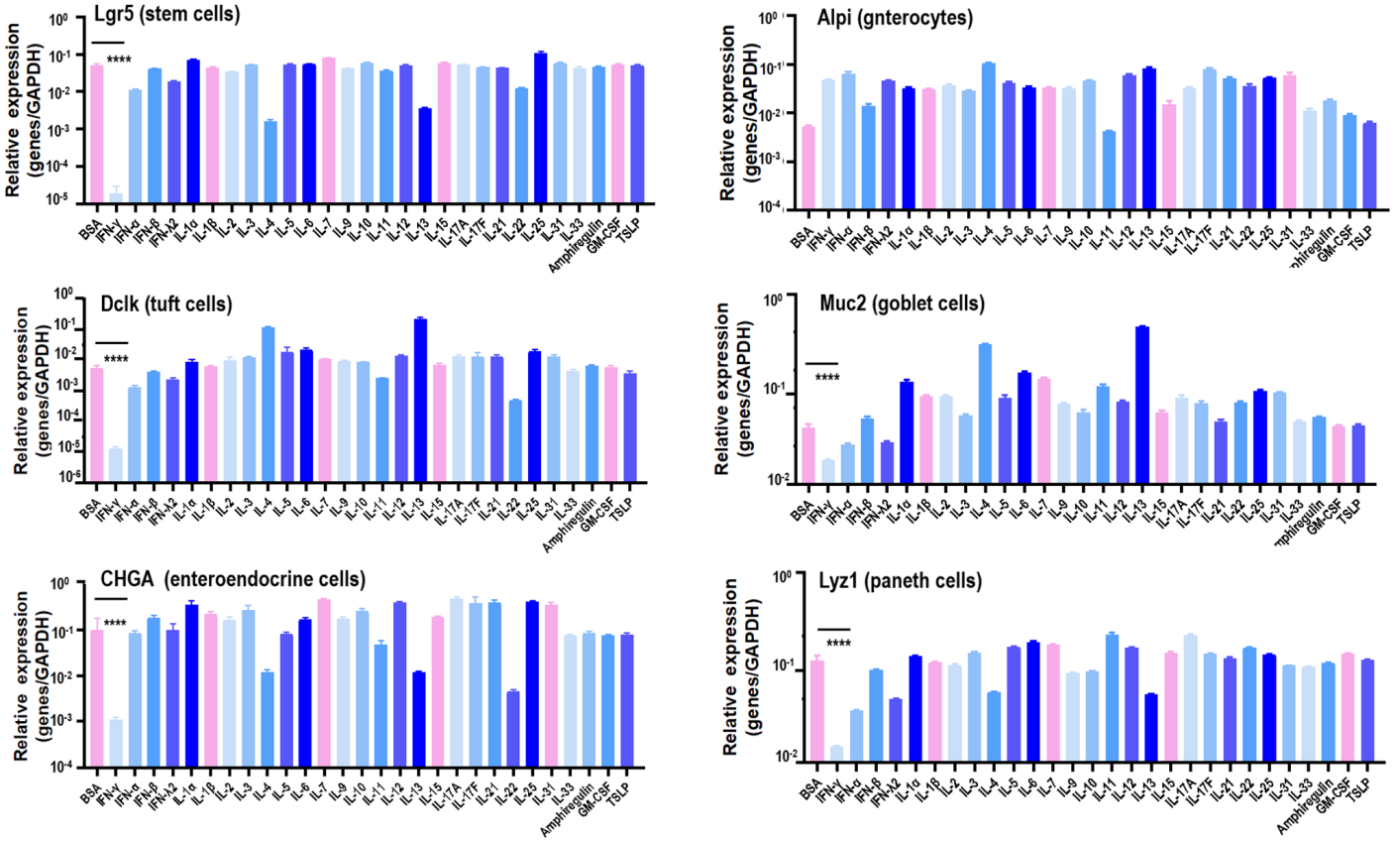
**Canonical marker gene expression in stem cells, enterocytes, Tuft cells, Goblet cells, enteroendocrine cells and Paneth cells in response to different cytokines.** Lgr5, Alpi, Dclk1, Muc2, CHGA, Lyz1 are marker genes of stem cells, enterocytes, Tuft cells, Goblet cells, enteroendocrine cells and Paneth cells, respectively.

Paneth cells, together with Tuft cells (epithelial chemosensory cells that can detect and relay information from diverse luminal substances via immune cells [17]), Goblet cells (specialized epithelial cells secreting pro-inflammatory cytokines [18]), enteroendocrine cells (chemo-sensors in the intestinal epithelium [19]), and enterocytes (the most abundant epithelial cell lineage in SI and LI that produces cytokines coordinating responses from subepithelial immune populations [20]), represent the principal types of cells in the SI epithelium [21]. To identify the specific cytokine that suppresses SI spheroids, the effects of a diversified spectrum of cytokines (IL-1α, IL-1β, IL-2, IL-3, IL-4, IL-5, IL-6, IL-7, IL-9, IL-10, IL-11, IL-12, IL-13, IL-15, IL17A, IL17F, IL21, IL22, IL25, IL31, IL33, IFNα, IFNβ, IFNγ, IFNλ2), thymic stromal lymphopoietin (TSLP) and other inflammation-associated factors such as amphiregulin (AREG, a ligand of EGFR) and granulocyte-macrophage colony stimulating factor (GM-CSF) on these primary SI epithelium cells were examined using the whole colorectal organoid. Stem cells (*Lgr5+*), Tuft cells (*Dclk1+*), Goblet cells (*Muc2+*), enteroendocrine cells (*GHGA+*), and Paneth cells (*Lyz1+*) expressed sufficiently lower levels of IFNγ than other cytokines examined, suggestive of the critical role of IFNγ in maintaining enteric homeostasis under physiological conditions as well as its suppressive functionalities on colorectal spheroid formation (Figure 1, Supplementary Figure 2).

### IFNγ suppresses SI spheroid growth via the IFNγ/IFNGR2/APC axis

**Figure 2:**
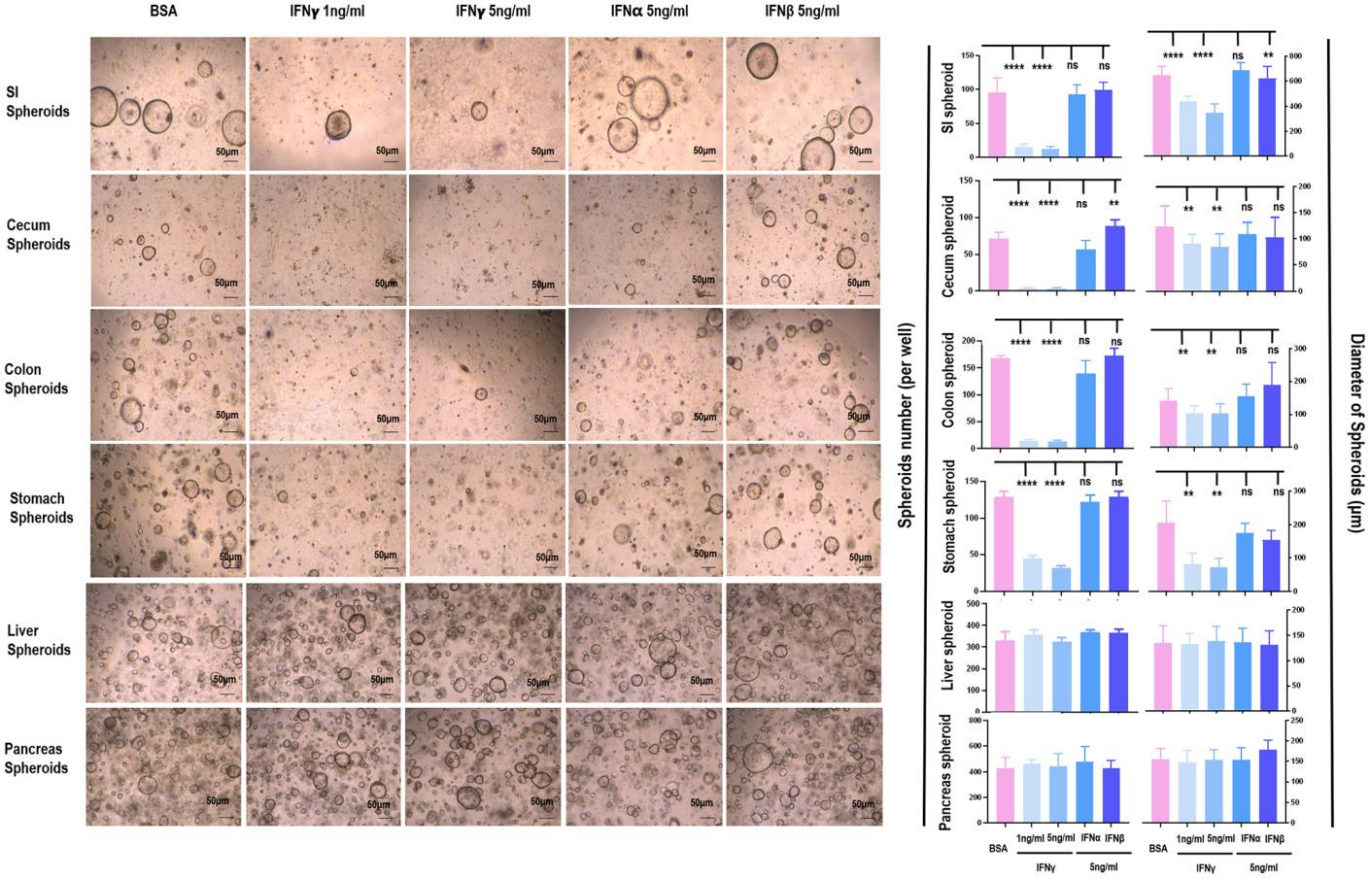
**The differential role of IFNα/β/γ on the proliferation of spheroids from different tissues.** The effects of IFNα/β/γ were examined where 1 ng/mL and 5 ng/mL of IFNγ, 5 ng/mL of IFNα, and 5 ng/mL of IFNβ were used, and BSA was used as the control.

**Figure 3:**
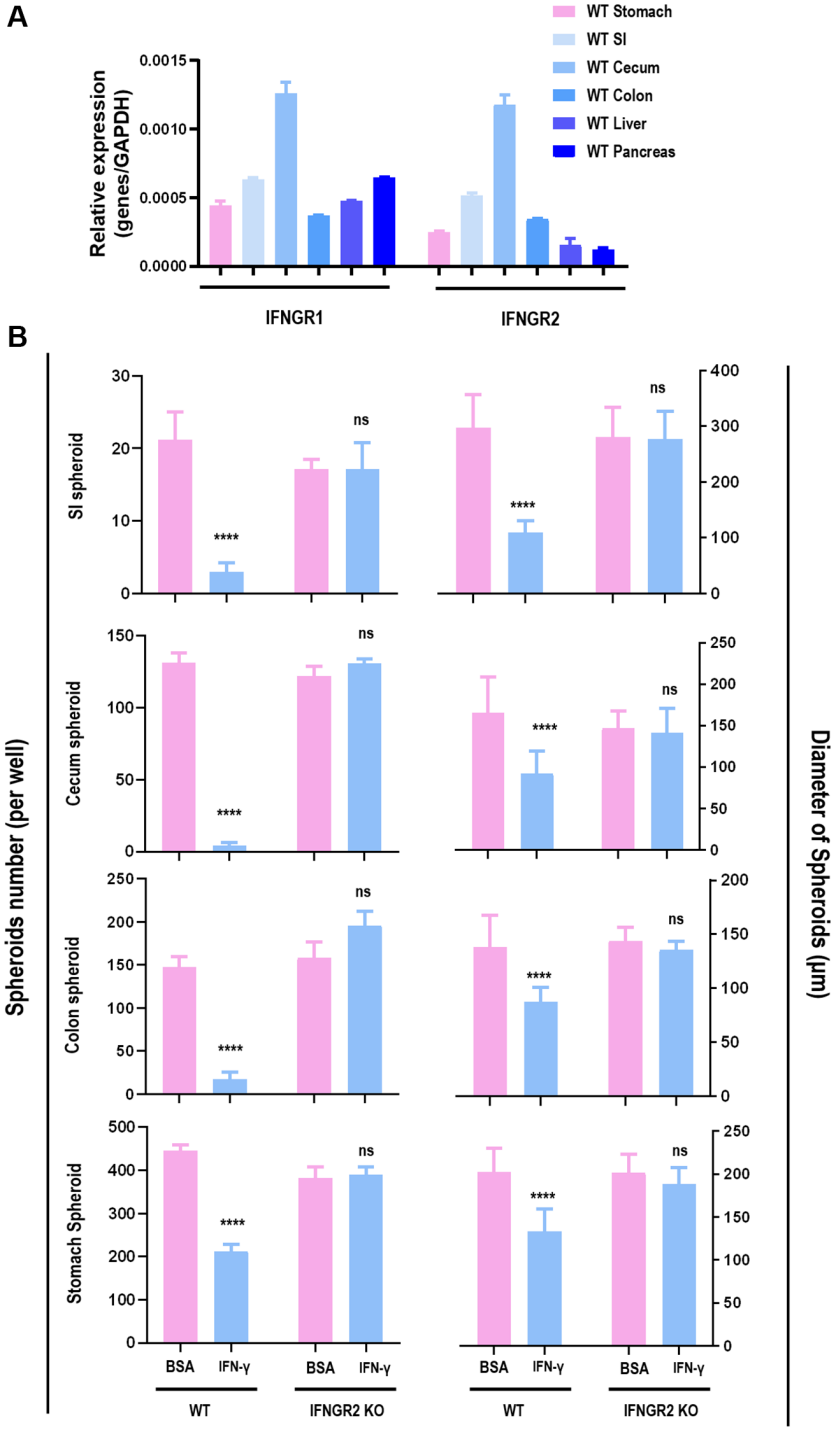
**The role of IFNGR2 in mediating the suppressive role of IFNγ on spheroid proliferation.** (**A**) *IFNGR1/2* expression in SI, stomach, cecum, colon, liver and pancreas. (**B**) The effect of knocking out *IFNGR2* on the amount and diameter of spheroids from SI, stomach, cecum, and colon.

IFNγ effectively reduced the amount and size of spheroids originated from SI (*p* < 0.001), cecum (*p* < 0.001), colon (*p* < 0.001), and stomach (*p* < 0.001), but not from liver and pancreas (Figure 2, Supplementary Figures 3, 4). Since the organoids were most sensitive to IFNγ treatment, and morphologies of these organoids were normal even under 5 ng/ml IFNγ treatment (as visualized in Figure 2), samples collected for RT-PCR were considered healthy. The RT-PCR results revealed substantially lower level of IFNGR2 but not IFNGR1 in liver and pancreas spheroids as compared with those of SI, cecum, colon, stomach spheroids (Figure 3A). Knocking out *IFNGR2* rendered cells irresponsive to IFNγ treatment in spheroids of SI, cecum, colon and stomach (Figure 3B, Supplementary Figure 5), suggestive of the role of the IFNγ/IFNGR2 axis in mediating the observed detrimental effects on SI spheroids as well as those from cecum, colon and stomach.

**Figure 4:**
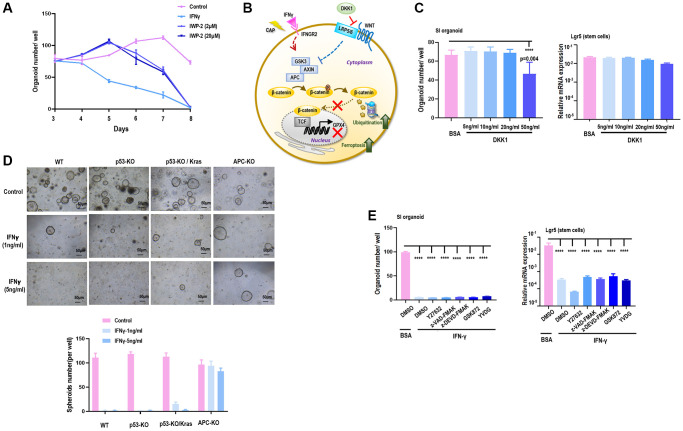
**APC is a target of IFNγ in suppressing spheroid proliferation.** (**A**) SI organoid amount under treatment of IFNγ, and Notch signaling inhibitor IWP2 at different concentrations (2 μM, 20 μM). (**B**) Hypothesized network explaining IFNγ triggered GPX4-dependent ferroptosis. (**C**) SI organoid amount and Lgr5 (stem cell marker) gene expression under treatment of Wnt signaling inhibitor DKK1 at different concentrations (5 ng/mL, 10 ng/mL, 20 ng/mL, 50 ng/mL). (**D**) SI organoid amount and Lgr5 (stem cell marker) gene expression under treatment of ROCK inhibitor Y27632, apoptosis inhibitor Z-VAD-FMK and Z-DEVD-FMK, necrosis inhibitor GSK872, cell cycle checkpoint inhibitor YVDG. (**E**) SI organoid amount and quantification in response to IFNγ treatment (1 ng/mL, 5 ng/mL) when p53, p53/Kras, APC were knocked out respectively.

Treating SI cells with Notch signaling inhibitor (IWP-2) under different doses (2 μM, 20 μM) did not exhibit similar time series with cells treated by IFNγ (Figure 4A) regarding the amount of SI organoid, suggesting that IFNγ/IFNGR2-mediated organoid reduction was not Notch-dependent.

Supplementing SI cells with DKK1, a Wnt signaling inhibitor that binds LRP6 and thus blocks interactions between LRP5/6 and Frizzled [22] (Figure 4B), under varied doses (5 ng, 10 ng, 20 ng, 50 ng) did not alter the amount of SI organoids nor Lgr5 expression (Figure 4C), suggesting that SI organoid reduction was independent of the binding between LRP5/6 and Frizzled in Wnt signaling. Next, we constructed SI cells carrying deficient *p53* and over-activated *Kras* (p53-KO/Kras), SI cells with *p53* being knocked out (p53-KO), and SI cells lacking *APC* expression (APC-KO) (Supplementary Figure 6). By supplementing these genetically modulated cells with 1 ng/ml or 5 ng/ml IFNγ, IFNγ significantly reduced the amount of SI spheroids when *p53* was knocked out independent of the status of Kras, but such a reduction was observed when *APC* was knocked out (Figure 4D), suggestive of the involvement of APC in IFNγ/IFNGR2-mediated organoid damage. APC is a multi-functional tumor suppressor gene that suppresses the canonical Wnt pathway via forming the complex with AXIN and GSK3, where the APC-AXIN-GSK3 complex inhibits Wnt signaling via promoting β-catenin ubiquitination [23]. These evidences altogether suggest that IFNγ/IFNGR2 may act through maintaining a persistent formation of APC with AXIN and GSK3.

Lastly, we accessed whether IFNγ/IFNGR2-mediated SI organoid damage was due to apoptosis (inhibitors: Z-VAD-FMK, Z-DEVD-FMK), necrosis (inhibitor: GSK872), akoisis (inhibitor: Y27632), cell cycle arrest (inhibitor: YVDG) by inhibiting each of these pathways, and find neither of these inhibitors reduced SI organoids nor rescued the reduced expression of Lgr5 when being subjected to IFNγ treatment (Figure 4E). This implicates that IFNγ/IFNGR2-mediated organoid damage was not due to apoptosis, necrosis, akoisis, or cell cycle arrest. It was lately reported that the Wnt/β-catenin axis suppressed ferroptosis in gastric cancer cells via targeting GPX4 [24]. Thus, it is highly possible that the observed SI organoid damage was a consequence of ferroptosis and GPX4 was a downstream target of β-catenin.

### IFNγ may synergize with CAP in suppressing SI spheroid growth via IFNγ/IFNGR2/APC/TCF/GPX4 signaling

Cold atmospheric plasma (CAP) is an emerging onco-therapeutic approach for cancer treatment as well as many other diseases [25]. Its efficacy against malignant cells has been demonstrated in various malignant cells such as colorectal cancers [26], liver cancers [27], stomach cancers [28], pancreatic cancers [29], and has been shown capable of damaging cancer stem cells [30].

**Figure 5:**
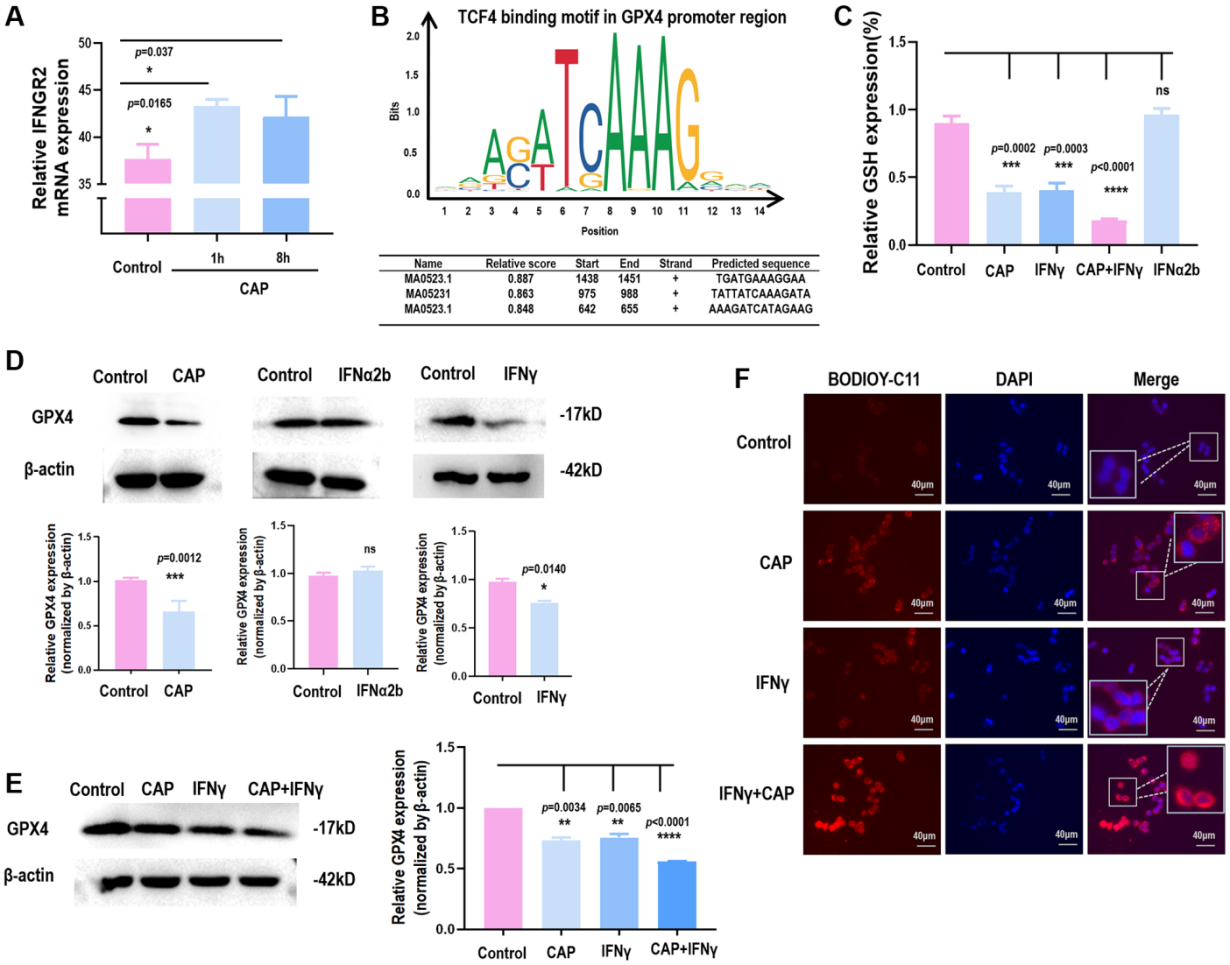
**CAP triggers GPX4-dependnet ferroptosis and creates synergies with IFNγ.** (**A**) IFNGR2 mRNA expression in response to CAP treatment under different exposure duration. (**B**) TCF4 is a potential transcription factor of GPX4 as predicted using the transcription factor binding prediction tool JASPAR [36]. (**C**) GPX4 protein level in response to IFNγ or IFNα. (**D**) Relative GSH level in response to CAP, IFNγ, IFNα, or ‘CAP plus IFNγ’. (**E**) GPX4 protein level in response to CAP, IFNγ, or ‘CAP plus IFNγ’. (**F**) Relative lipid peroxidation level in response to CAP, IFNγ, or ‘CAP plus IFNγ’. Data from panel A were performed using SUM159PT cells, and panels C to F were performed using the HCT8 cell line.

We have previously shown that CAP can selectively arrest the growth of triple negative breast cancer (TNBC) cells [31], the subtype with the highest stemness among all breast cancers [32]. By comparing the full transcriptome of TNBC cells with and without receiving CAP exposure for different durations, IFNGR2 was found significantly increased in response to CAP treatment (Figure 5A), suggestive of IFNγ signaling activation and SI organoid damage after CAP treatment.

Several studies have reported the role of CAP in inducing cancer cell ferroptosis [33–35], consolidating our hypothesis on the regulatory role of β-catenin on GPX4 through forming complexes with TCF. Through transcription factor binding site prediction using JASPAR [36], TCF4, a member of TCF family, was found to transcriptionally bind to the promoter region of GPX4 (Figure 5B). In the canonical Wnt pathway, the activity of β-catenin is predominantly mediated by TCF4, a member of the TCF family transcription factors [37]. In addition, we found that either CAP or IFNγ reduced GPX4 protein expression (Figure 5C) and GSH level (Figure 5D), but the same effect was not observed when IFNα was used (Figure 5C, 5D). IFNγ created synergy with CAP in reducing GSH level (Figure 5D), GPX4 protein expression (Figure 5E), and lipid peroxidation (Figure 5F), suggestive of their synergized efficacy in triggering GPX4-dependent ferroptosis.

## DISCUSSION

IFNγ suppressed the amount and size of spheroids originated from SI, cecum, colon, and stomach, but not from liver and pancreas (Figure 2, Supplementary Figure 3), suggestive of its specificity against gastrointestinal malignancies. Also, among all interferons examined, only IFNγ showed the desired efficacy. While IFNα and IFNβ are type I interferons and IFNλ2 belongs to the type III family, IFNγ is the sole interferon type II family member. Though type I interferons such as IFNα1b and IFNα2b have been widely used in clinics for cancer treatment, IFNγ (a type II interferon) has not been clinically administrated. Besides, though there have been several clinical trials examining the efficacy and safety of IFNγ as an onco-therapy along with other treatment modalities (Supplementary Table 6), no trial has been initiated to investigate the efficacy or safety of IFNγ as an anti-cancer therapy alone. In addition, only 1/4 of these clinical efforts (NCT03112590, NCT02948426, NCT00786643, NCT00501644, NCT01881867, NCT00266110, NCT01957709, NCT00821964) have results available, with NCT00786643 being the only one administrated to colorectal cancer patients. In NCT00786643, IFNγ was used together with 5-fluorouracil, leucovorin, bevacizumab, where only 5 out of 20 patients showed partial response (≥30% decrease in sum of longest diameter of target lesions) to this treatment regimen, and 10% and 18.52% patients from stratum 1 and stratum 2 showed serious adverse responses, respectively. Thus, there has not been strong clinical evidence to support the use of IFNγ in cancer treatment. Our results reported the unique feature of IFNγ in treating gastrointestinal cancers, demonstrating its feasibility in being applied in oncology.

We used SI spheroids as the primary model in this study. This experimental design can not only identify cytokines capable of suppressing cancer stemness but also characterize those with critical roles in maintaining enteric homeostasis, given the fact that colorectal CSCs are transformed ISCs. This implicates the seemingly paradoxical functions of IFNγ under physiological and pathological conditions, and emphasizes its careful use in treating different diseases. For instance, IFNγ may increase the risk of developing inflammatory bowel diseases by impairing Paneth cell viability and jeopardizing enteric homeostasis [38], despite its beneficial roles in suppressing colorectal CSCs and arresting cancer cell proliferation.

We investigated whether IFNγ-induced SI spheroid suppression was mediated by Wnt or Notch signalings, as they are known pathways regulating colorectal CSCs [6]. In addition, we constructed *APC*, *p53*, *Kras* mutants to explore key players driving the IFNγ/IFNGR2 axis given that they are common genetic mutations in colorectal cancers influencing CSC dynamics during tumor initiation [6]. Consequently, we narrowed down our focus to Wnt signaling and APC. Using DKK1 as the Wnt inhibitor, we excluded ‘interactions between LRP5/6 and Frizzled’ (the initiation phase of Wnt signaling) from the IFNγ/IFNGR2 axis. Using the *APC*-knockout mutant, we unveiled the involvement of ‘the complex formed among APC, AXIN and GSK3’ in the IFNγ/IFNGR2 axis. Approximately 80–85% sporadic colorectal cancers carry an *APC* mutation that results in activated β-catenin signaling and enhanced Wnt target expression [39, 40]. These together suggest that although IFNγ can target colorectal cancer stemness, it may hold true for only a small proportion of sporadic colorectal cancer patients, and additional strategies are needed to enhance the sensitivity of colorectal cancer cells carrying *APC* mutation to IFNγ treatment.

Enhanced ROS levels at the crypt base are known to play an important yet unknown role in regulating both ISCs and colorectal CSCs [6], suggesting that redox strategies may function through IFNγ/IFNGR2 signaling. In consistent with this, ionizing radiation was shown capable of resolving the tumor burden in wildtype mice, but did not arrest tumor growth when the gene encoding IFNγ was knocked out using a murine colorectal cancer model [41].

CAP is an emerging onco-therapeutic strategy relying on redox control. Its selectivity against cancer cells, i.e., selectively killing transformed cells without affecting the growth of their healthy peers, has been demonstrated in various tumor models [25]. Though consecutive efforts have been devoted to uncover the underlying molecular mechanism driving its anti-cancer capacity, the signaling roadmap is still far from being complete given its multi-modality as a result of content complexity and dose-dependent nature. We have previously shown that CAP can arrest TNBC stemness via modulating AQP3-19Y mediated AQP3-5K and FOXO1 K48-ubiquitination [30]. By analyzing the whole transcriptome data obtained using TNBC cells, we found that CAP significantly elevated the expression of *IFNGR2* (Figure 5A), which not only consolidated our previous conclusion but also implicated an alternative pathway mediating CAP-triggered inhibition on TNBC stem cells, i.e., via the IFNγ/IFNGR2/APC axis. Importantly, these results may suggest a beneficial synergy between IFNγ and CAP in treating colorectal cancers and possibly other forms of malignancy as well. That is, although IFNγ is not an ideal remedy for treating colorectal cancers, CAP can sensitize tumor cells to IFNγ treatment.

We also investigated the cell death programs underlying the observed suppression on SI organoids using inhibitors of apoptosis, anoikis, necrosis, and cell cycle checkpoints. Though none of these inhibitors rescued the suppression of IFNγ on SI organoids, we found that TCF4, the interactor of β-catenin, is the transcription factor of GPX4 (Figure 5B), a canonical marker of ferroptosis. Consistent with this, TCF was proposed as the transcription factor of GPX4 in gastric cancers [24]. Thus, it is worth to verify experimentally whether the IFNγ/IFNGR2/APC/TCF4/GPX4 axis exists in colorectal cancer cells that, once taking on actions, triggers ferroptosis in colorectal CSCs. These are not covered in this study and left for future investigations.

## CONCLUSIONS

We, in this study, identified the critical role of IFNγ in arresting SI organoids via the IFNγ/IFNGR2/APC/TCF4/GPX4 axis, and proposed its efficacy in arresting colorectal CSCs via possibly triggering GPX4-dependent ferroptosis. Our results provide the first-hand data supporting the benefits of using IFNγ, and especially when combined with CAP, for colorectal cancer treatment that not only opens a novel avenue for the clinical use of IFNγ but also broadens our understandings on the anti-cancer mechanism of CAP for clinical translation.

## Supplementary Materials

Supplementary Figures

Supplementary Tables
