# Supplementary material for: IFNγ synergies with cold atmospheric plasma in triggering colorectal cancer cell ferroptosis via the IFNγ/IFNR2/APC/TCF4/GPX4 axis
Source: Aging (Albany NY). 2023 Sep 4;15(17):8692–711. doi: 10.18632/aging.204985 (PMC10522381; doi:10.18632/aging.204985)
Supplement: Supplementary Figures [file aging-15-204985-s001.pdf]

SUPPLEMENTARY FIGURES

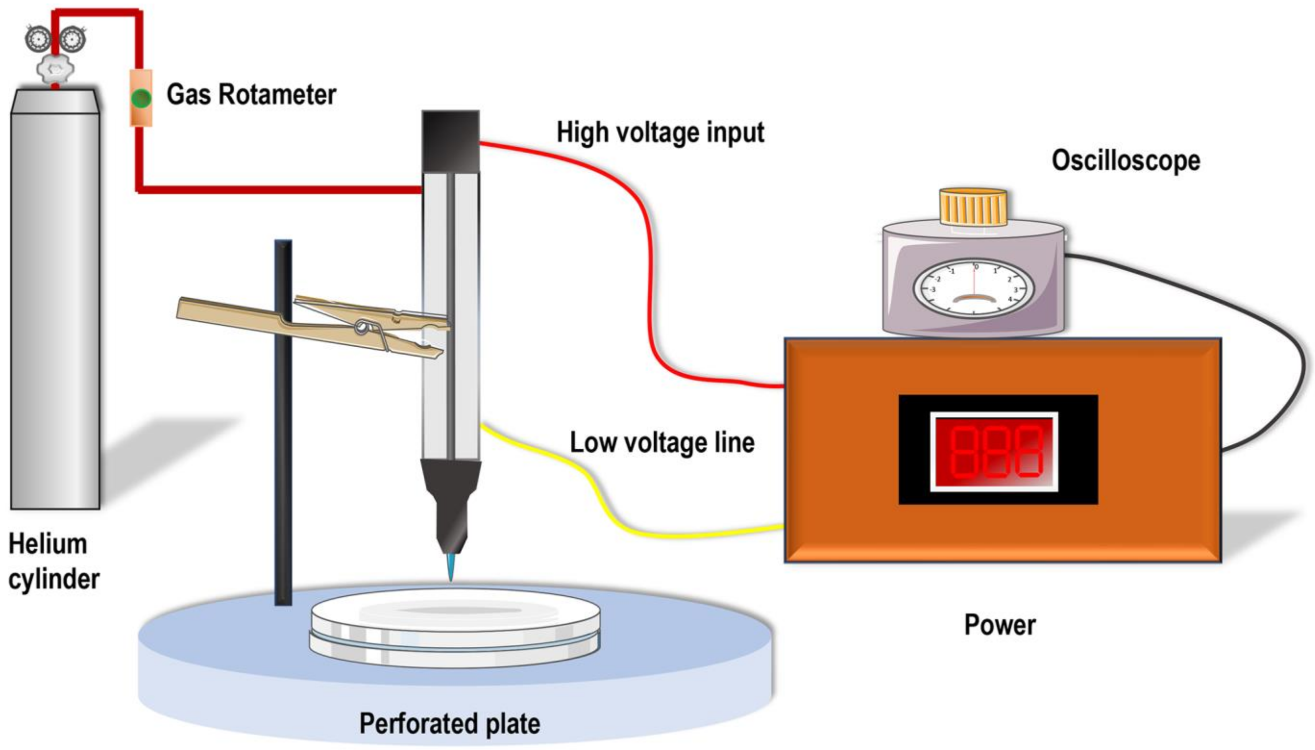

Supplementary Figure 1. Schematic diagram showing the equipment for CAP ejection.

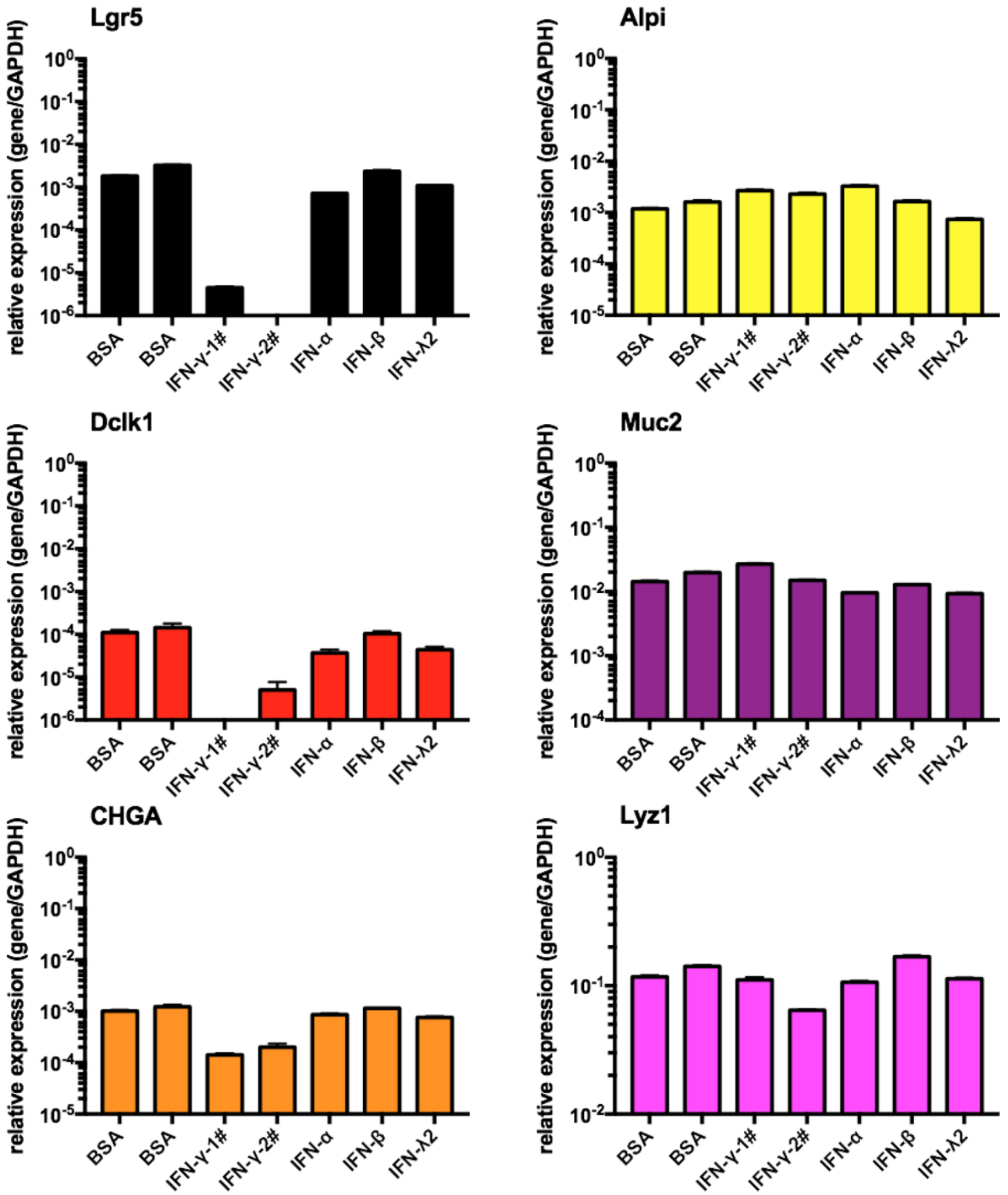

Supplementary Figure 2. Canonical marker gene expression in stem cells, enterocytes, Tuft cells, Goblet cells, enteroendocrine cells and Paneth cells in response to IFNα/β/γ/λ2. IFNγ-1, IFNγ-2 were purchased from Abisin (#abs04123) and Yeasen (#91211ES10), respectively.

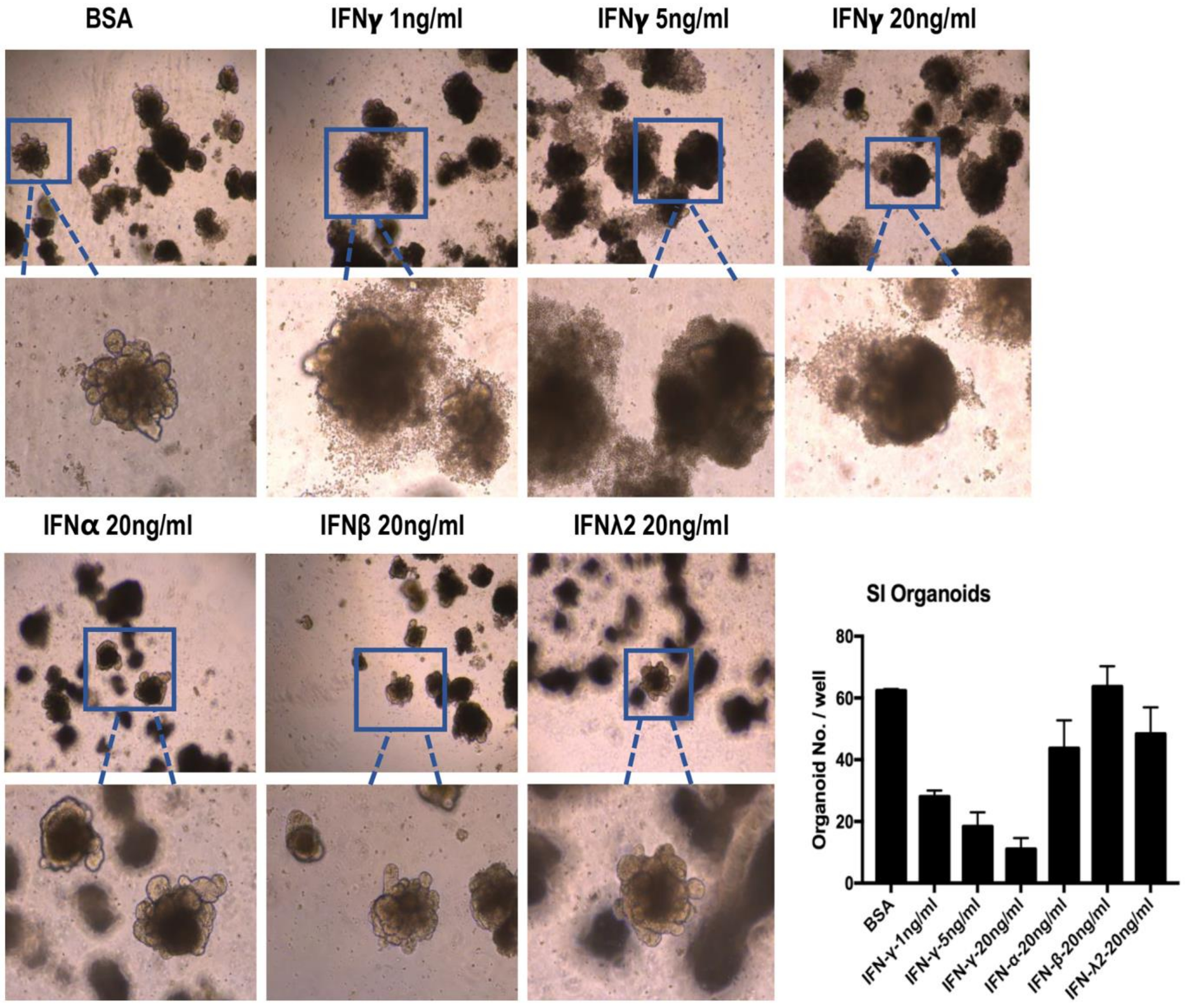

Supplementary Figure 3. The amount of SI spheroids in response to IFNα/β/γ under different concentrations.

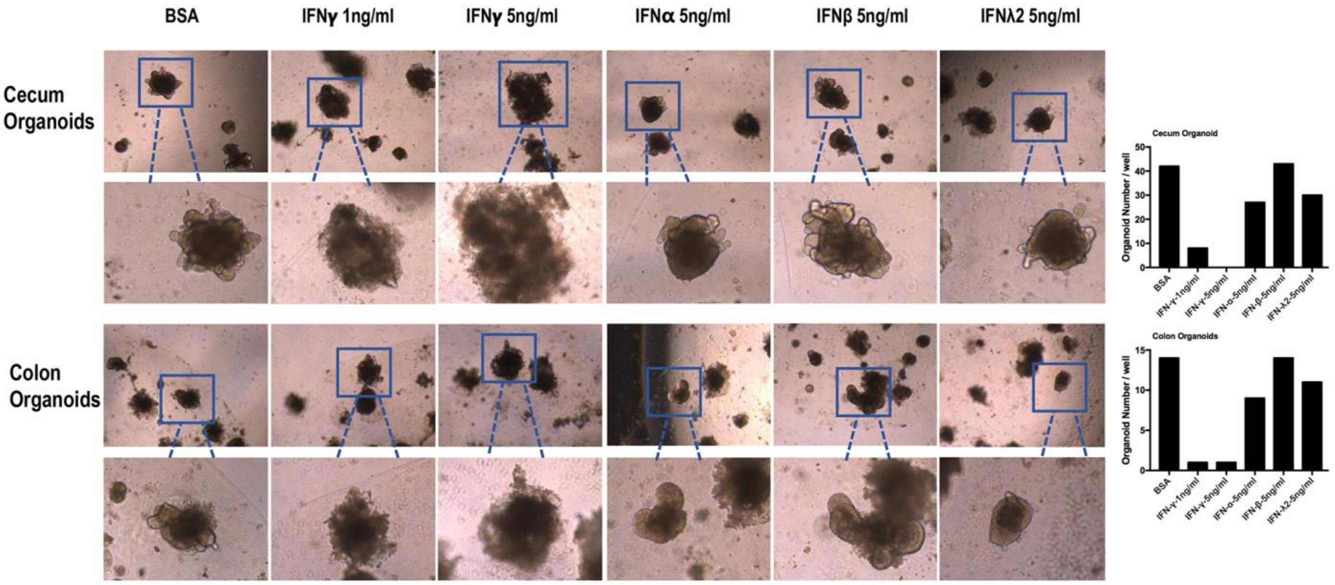

Supplementary Figure 4. The amount of cecum and colon spheroids in response to IFNα/β/γ under different concentrations.

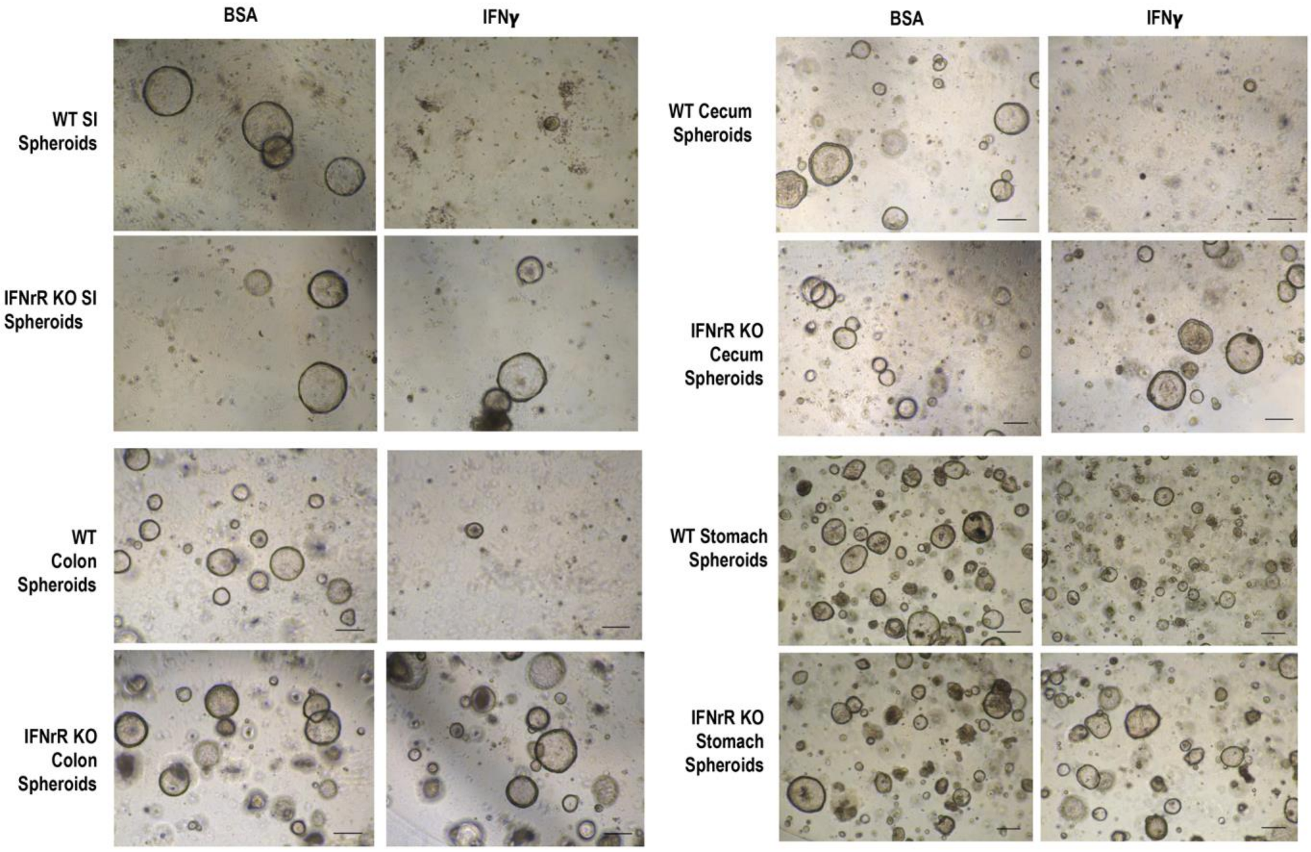

Supplementary Figure 5. Images of spheroids from different tissues in response to IFNγ when knocking down *IFNGR1/2*.

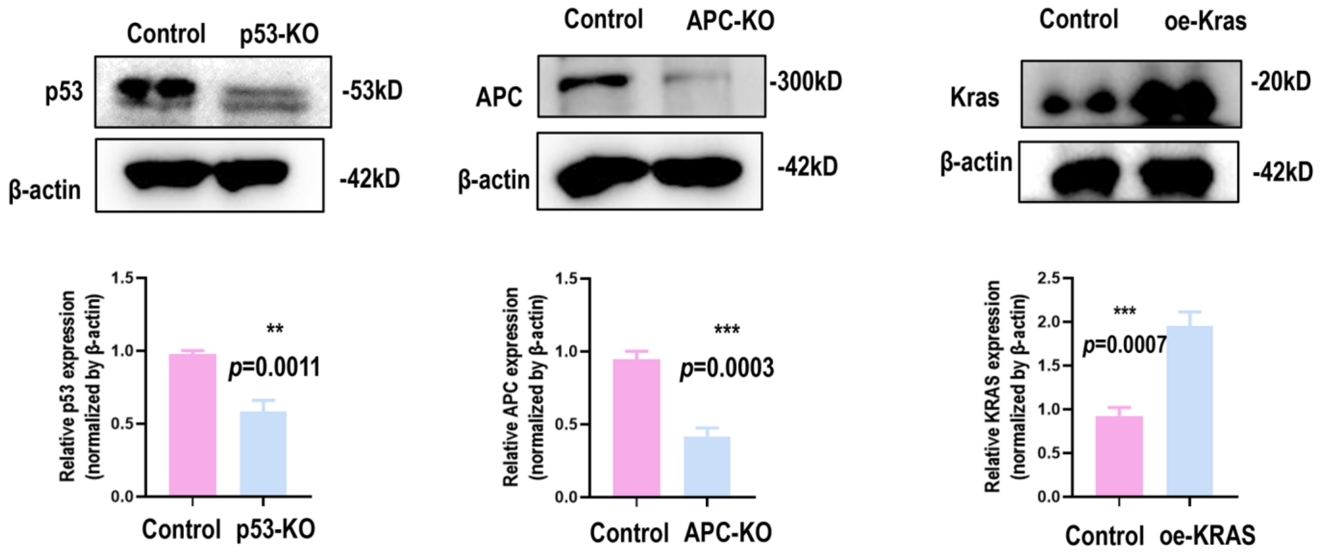

Supplementary Figure 6. The knockout of *p53*, APC or the hyperactivation of Kras.
