# Supplementary material for: IFNγ synergies with cold atmospheric plasma in triggering colorectal cancer cell ferroptosis via the IFNγ/IFNR2/APC/TCF4/GPX4 axis
Source: Aging (Albany NY). 2023 Sep 4;15(17):8692–711. doi: 10.18632/aging.204985 (PMC10522381; doi:10.18632/aging.204985)
Supplement: Supplementary Tables [file aging-15-204985-s002.pdf]

SUPPLEMENTARY TABLES

Supplementary Table 1. Information on the media and supplied cytokines used for organoid culturing.

Organoid name	Culture medium catalog No. and company	Additional cytokines
SI Organoid	#355357, Corning	EGF, Noggin, R-Spondin 1, Wnt-3a
Cecum Organoid	#355357. Corning	EGF, Noggin, R-Spondin 1, Wnt-3a
Colon Organoid	#355357, Corning	EGF, Noggin, R-Spondin 1, Wnt-3a
Stomach Organoid	#CM-H048, Pricella	EGF, Noggin, R-Spondin 1, FGF-10, BMP-4
Liver Organoid	#355056, Corning	BMP-4, EGF, FGF-basic, FGF-10, HGF, Noggin, Wnt-3a
Pancreas Organoid	#CM-H020, Pricella	EGF, Noggin, R-Spondin 1, Wnt-3a, FGF-10

Supplementary Table 2. Sequences of primers used in this study.

Gene name	Forward (5′–3′)	Reverse (5′–3′)
Lgr5	CTCCCAGGTCTGGTGTGTTG	GAGGTCTAGGTAGGAGGTGAAG
Alpi	CGACCGGGCGATTCAGAGAG	GTAATACGACTCACTATAGGGTGGTGTACGGCTCAA
LYZ1	GGGAACCTGTGACCTGTCTT	GCCTCATGACACTGGGAACA
Dclk	CAGCCGCAATTCAATCAGTA	GCAACTGTGAGTGGCTCTGA
CHGA	TGTATCGTCGAGGTCATCTCTGA	CGAGGTCTTGGAGCTCTTTCA
IFNgR1	AGTGCTTAGCCTGGTATTCATCTG	GGCTGGTATGACGTGATGAGTG
IFNgR2	CAGGAGCCTGTTTCTTCCTG	TTCCGGAAACGAGATAATGG
GAPDH	TGCACCACCAACTGCTTAGC	GGCATGGACTGTGGTCATGAG

Supplementary Table 3. Information on cytokines and other reagents used in this study.

Reagent name	Catalog no.	Company
BSA	#HY-D0842	MCE
IFNγ	#abs04123	Abisin
IFNα	#abs06261	Abisin
IFN-β	#abs000916	Abisin
IFNλ2	#abs01022	Abisin
IL-1α	#abs00801	Abisin
IL-1β	#abs00802	Abisin
IL-2	#abs00804	Abisin
IL-3	#abs00805	Abisin
IL-4	#abs00806	Abisin
IL-5	#abs00807	Abisin
IL-6	#abs00808	Abisin
IL-7	#abs00809	Abisin
IL-9	#abs00812	Abisin
IL-10	#abs00813	Abisin
IL-11	#abs00814	Abisin
IL-12	#abs00815	Abisin
IL-13	#abs00816	Abisin
IL-15	#abs00818	Abisin
IL-17A	#abs04047	Abisin
IL-17F	#abs00823	Abisin
IL-21	#abs05146	Abisin

IL-22	#abs00827	Abisin
IL-25	#abs00984	Abisin
IL-31	#abs00828	Abisin
IL-33	#abs00829	Abisin
Amphiregulin	#abs00904	Abisin
GM-CSF	#abs01044	Abisin
IFNβ	#abs00916	Abisin
TSLP	#abs00844	Abisin

Supplementary Table 4. Information on inhibitors used in this study.

Inhibitor name	Pathway inhibited	Catalog no.	Company
DKK1	Wnt pathway	#P02228	Solarbio
IWP-2	Notch pathway	#HY-13912	MedChemExpress
Y27632	Rho-kinase, anoikis	#07171	StemCell™ Technologies
z-VAD-FMK	Apoptosis	#HY-HY-16658B	MedChemExpress
z-DEVD-FMK	Apoptosis	#HY-12466	MedChemExpress
GSK872	Necrosis	#HY-101872	MedChemExpress
YVDG	Cell cycle	#HY-16990	MedChemExpress

Supplementary Table 5. Information on antibodies used in this study.

Antibody name	Catalog no.	Company	Dilution ratio
GPX4	#67763-1-Ig	Proteintech	1: 1000
APC	19782-1-AP	Proteintech	1:1000
p53	# 2524S	Cell Signaling Technology	1:1000
KRAS	12063-1-AP	Proteintech	1:1000
β-actin	#PTM-5018	PTM-BIO	1: 2000
HRP labeled Goat anti Rabbit IgG	#A0208	Beyotime	1: 5000
HRP labeled Goat anti Mouse IgG	#A0216	Beyotime	1: 5000

Supplementary Table 6. Current status on clinical trials using IFNγ for cancer treatment.

NCT no.	Status	Results available	Cancer type	Phase
NCT03112590	Completed	Yes	Breast Cancer	Phase 1|Phase 2
NCT02948426	Terminated	Yes	Ovarian Cancer	Phase 1
NCT00004032	Completed	No	Ovarian Cancer	Phase 1
NCT02614456	Completed	No	Advanced Solid Tumors	Phase 1
NCT00002637	Completed	No	Prostate Cancer	Phase 1|Phase 2
NCT04540224	Completed	No	Breast Cancer	Not Applicable
NCT00002796	Terminated	No	Colon Cancer	Phase 1|Phase 2
NCT00786643	Completed	Yes	Colorectal Cancer	Phase 2
NCT00004016	Completed	No	Melanoma	Phase 1
NCT00501644	Completed	Yes	Ovarian Cancer	Phase 2
NCT00047632	Terminated	No	Ovarian Cancer	Phase 3
NCT02016833	Completed	No	Ovarian Cancer	Not Applicable
NCT03782428	Completed	No	Colorectal Cancer	Not Applicable
NCT00001296	Completed	No	Melanoma	Phase 3

NCT01461148	Completed	No	Colorectal Cancer	Phase 1|Phase 2
NCT01881867	Completed	Yes	Prostate Cancer	Phase 2
NCT00266110	Completed	Yes	Breast Cancer	Phase 2
NCT03005860	Unknown	No	Breast Cancer	Not Applicable
NCT04438564	Recruiting	No	Breast Cancer, Colorectal Cancer	Not Applicable
NCT00008203	Completed	No	Breast Cancer	Phase 3
NCT02197169	Completed	No	Glioblastoma, Gliosarcoma	Phase 1
NCT00059878	Completed	No	Solid Tumor	Phase 2
NCT00616720	Completed	No	Multiple Myeloma	Phase 2
NCT00002505	Completed	No	Adult Solid Tumor	Phase 2
NCT00428272	Terminated	No	Osteosarcoma	Phase 1
NCT00844506	Completed	No	Ovarian Cancer	Phase 2
NCT01957709	Terminated	Yes	Liposarcoma	Phase 1
NCT03331367	Completed	No	Prostate Cancer	Not Applicable
NCT00656123	Completed	No	Colorectal Cancer	Phase 1
NCT00821964	Completed	Yes	Breast Cancer	Phase 2
NCT04864379	Recruiting	No	Advanced Solid Tumor	Phase 1
NCT04328714	Recruiting	No	Acute Leukemia	Phase 1
